# Observation Abroad

**Published:** 1892-06

**Authors:** Thomas M. McIntosh

**Affiliations:** Thomasville, Ga.


### OBSERVATIONS ABROAD.

THOMASVILLE, GA., May, 1892.

*Editors Southern Medical Record—*

A few observations while in Vienna recently may be of interest to your readers now. The statment is made there by Fenger, a genito-urinary specialist, that 43 per cent. of the sterilities is a fault in the male, and that 70 per cent. of this 43 is caused by gonorrhœa and its sequences, which he claims is never cured but exists in a latent form, capable of producing infection. The sterility is due to two conditions: asperma, when spermatozoa are absent; necro-sperma, when they are present, but dead. Asperma exists when from epididymitis or orchitis there is a permanent hypertrophy or a secondary atrophy of the testes, or when there is an inflammatory closure of the seminal ducts without orchitis or epididymitis.

In necro-sperma the sperm forming glands and ducts remain normal, but there is a secretion of pus, especially in the prostatic portion of the urethra. The normal reaction of the prostatic fluid is acid, due to phosphoric acid; the reaction of pus is alkaline. The spermatozoa normally do not manifest evidences of life, or have motion until they are immersed in the prostatic secretion, which in chronic gonorrhœa is alkaline by reason of the pus, and fails to vivify the semen, and thus the dead spermatozoa. These results of gonorrhœa in men will relieve many a woman from the fault of sterility usually attributed to her, and take many patients from the hands of the gynæcologist who might be consulted because of sterile wedded life. However, that a gonorrhœa is never cured and retains indefinitely its power of infection, will not generally be accepted, for there is no anatomical or histological reason why a specific urethritis should remain uncured that would not apply to other mucous membranes specifically inflamed; and there is the greater one of every-day observation, that 80 per cent. or more of the men have a gonorrhœa before marriage, who beget children, and whose wives are healthy. There can be no doubt, however, that many men are sterile from the complications of gonorrhœa, producing permanent organic changes, and in this sense it is incurable. A number of years ago Noeggerath, then of New York, proclaimed as the etiology of all inflammations of the uterine mucous membranes and its appendages, a latent gonorrhœa. The opinion was so repugnant to the sentiment of the public and so contrary to views of established medical opinion and teaching, that he lost his practice and went to a little town in Germany. Now, any number of eminent authorities agree with him. Therefore, if the above is true as applied to both sexes, gonorrhœa is a very serious disease in both a clinical and social sense, and we must accept it as true in so far as we do have often serious and incurable complications of gonorrhœa in both sexes, though we do and must doubt the continual duration and infectious character, even though the microscope should find a gonococcus in ten or twenty years. If the gonococcus is pathognomonic of gonorrhœa, we can easier believe that frail humanity has "loved again, not wisely, but too often," and has a new infection, and that the disease has not existed for twenty years, latent and uncured.

Under the teaching of Dr. Grunfeld, who has written much on the subject, one becomes very much interested in the use of the endoscope in the diagnosis of diseased conditions of the mucous membrane of the male and female urethra, and convinced of its practical value in the treatment of same. I do not refer to cystoscopy, which requires electric light illumination and an expensive apparatus difficult to keep in order.

In acute gonorrhœa, he uses the usual method of injection, and uses the endoscope chiefly to diagnose those conditions which produce discharges for such a time after the acute stage has passed as to warrant the term "chronic gonorrhœa." Just when the chronic gonorrhœa begins and the acute ends, he does not definitely say, but usually after six weeks duration he thinks it is chronic. However, he does not wait this long before the use of the endoscope, but may do so as soon as the acute condition of swelling and pain has gone. From an endoscopic standpoint he divides urethritis into simple, blenorrhagic, membranous, phlyctenula and ulcerative, which last includes the gumma and chancroid in the urethra. The conditions correspond in appearance and histologically to the same situated in other mucous membranes, a membranous, phlyctenula or granula conjunctivitis, or the so-called dry catarrh of the nose and throat or a mucous placque or gumma. Often following a blenorrhœic urethritis, there is a development of a granular condition of the urethral mucuos membrane, which is best treated by the application of sulphate of copper, though not so often as in conjunctival granulation, because the epithelial regeneration is not so rapid in the urethra. Following this granulation is often the histological condition of atrophy of the mucous membrane, and the clinical one of dryness and an abnormally pale and glistening appearance. In addition to the atrophy of the mucous membrane, there may be the development of new tissue, (the fibrous tissue of stricture) and these are treated by the direct application of iodide potash and iodine, one part of each to sixty of glycerine. The ulcerations are treated as those of the same character would be elsewhere situated. Of course this treatment can be varied to suit the case and condition, and the preference of the physician. As an illustration of the diagnostic value of the instrument, there was a case which to the bougie gave the characteristics of the so-called elastic stricture. The endoscope showed a white bridge of tissue extending directly across the urethra from side to side, behind which a sharply curved probe was passed, the point of which coming in view, isolated in the curve of the probe, the bridge of tissue, and traction being made on the probe, clearly proved the diagnosis.

The endoscope is merely an urethral speculum, through which, with the reflected light of the mirror, one can plainly see every part of the urethra; diagnose and treat with exactness the part diseased, just as one does the vagina, nose and throat, and it can be just as easily done. One needs only five endoscopes, 1, eight millimetres long, number 25 French scale; 2 ten millimeters long, numbers 18 and 20, and 2 thirteen millimetres long, numbers 18 and 24. A long slender probe or wire, on the end of which a little absorbent cotton is wrapped to clean the parts, and to apply the medicament. I go into some details because the American authors speak very little of it, because I believe it is not much valued by them.

The surgeons there and in Berlin only know Otis' method of treating stricture in theory. The cutting of stricture is rare, very rare indeed. There are surgeons there of large experience who have, perhaps, never made an internal urethrotomy. The method is universally by gradual dilation, and the large numbers are not used. Number 28, French scale, being considered large. Billroth and his assistant, Eisilberg, perform many bold operations. In going through the wards, I saw a case upon May 23d, in which there had been a resection of almost the entire 2d, 3d, 4th, 5th, 6th, 7th and 8th rib, and the patient is doing well with an immense hole in his chest into which the fist could be thrust, and the respiratory movements of the lung are plainly visible. It was done for chronic empyema (upon which a partial resection of two ribs had been previously made without benefit) that the chest walls might retract, and the pleural surfaces approximate and the cavity be obliterated.

A case also of cancer of the rectum in which he had removed the entire rectum, almost the entire sacrum and all of the posterior vaginal wall, (the anterior vaginal wall and mouth of the womb were plainly visible, when the dressing was removed) and the patient was well after this extensive mutilation.

In the pathological room, Prof. Kalisko exhibited the internal viscera of a patient that had died from the "dropsy," dependant on cardiac disease. The mucous membrane of the intestines was intensely congested, and had numerous ulcerations quite through to the sub-mucous tissue. These ulcerations were due, he explained, to the administration of calomel, which, passing into the circulation, came in contact with the sulphur of the intestinal canal forming an escharotic mercuric salts. These ulcerations he had often noticed in cases of general œdema in which calomel had been given, but he said that they were of no importance, as they soon healed.

In the medical wards, I learned that all cases of "dropsy" not dependant on disease of the kidneys, were treated with 8 to 12 decigrams of calomel each day until there was a slight tenderness of the gums; this allowed to pass away, when calomel again was given and so on in repetition. It is not given where the kidneys are diseased, because they then fail to respond to its influence, because it is an irritant diuretic that aggravates the disease.

This classification of calomel among the diuretics and its use as such in the general œdemas, originated in Europe, and has been used some in New York. The writer has never tested the treatment because he has not thought it correct in theory as to the action of the remedy, or being so, would not be warranted by clinical results.

When he has given calomel in such cases, it has been upon the principles which would indicate it in any disease where there was a sluggish action of the entire elimnatory functions with a view chiefly to its action upon the liver and alimentary canal.

In such cases where the urine, previously red and scant, has become clear and free following its action, he has regarded it as a result of the free cleansing of the system, as it were, through the alimentary canal, as a result of which all of the organs resumed, to a more or less degree, their normal functions, in which the kidneys also participated secondarily, and which any good purgative would produce. But accepting the observation of these eminent men as to the direct and potent diuretic action of calomel, the treatment is yet not the best that can be given, as there are other remedies which are equally as good, and which produce little general depression, and no local lesion.

The treatment of dropsies due to organic causes, can only be symptomatic and palliative, the object being, by attention to diet and drink, to give the greatest amount of nutrition with the minimum of fluids, and to remove the œdema by those means which will the least depress the system or disturb the digestion.

Calomel is a remedy depressing in its effects when given in large and repeated doses, and to salivate a patient even in an acute inflammatory disease is not desired and is especially bad in one whose course is chronic and incurable. In such cases Glauber's salts, in doses from one to two tablespoonsful two or three times a week, (given before breakfast) has seemed to the writer to be of great value. There are usually four or five large watery actions with no depressing effect, and slight disturbance of the digestion, which sometimes gives such relief from the dyspnœa that the patient will request another dose.

Since, therefore, calomel produces ulcerations in the intestinal mucous membranes sometimes, it must surely at all times produce great irritation of it, and therefore is not a good remedy when so administered.

I have said nothing in other letters written you of Koch's lymph, because every one is so familiar with its history. It is yet used by some there for diagnostic purposes, or rather as one of the means of verification, and also sometimes as a therapeutic measure in the external forms of tuberculosis, in which however it has no advantage over other means of treatment or is not so good. In lupus, the old method of thoroughly curetting is more certain of prompt relief and it is just as permanent.

Koch, in a very recent publication, claims to have eliminated the fever producing element. This is much accomplished.

While speaking of lupus and tuberculosis, it may be perhaps interesting to know that Kaposi, the great authority on skin diseases, stands boldly alone in his adherence to the old idea of classifying these diseases as distinct pathologically. Since the discovery of the bacillus of tuberculosis in both diseases, and since the behavior of Koch's lymph is similar in both, pathologists have regarded them as one and the same process.

These observations Kaposi regards only as incidents in the action of a remedy, and the discoveries of the microscope, and believes the bacillus, not the "*proctor hoc*" of lupus. His reasons are based on the widely different clinical histories of the diseases.

Lupus is a disease of the skin and remains local, is long in its duration, sometimes existing for over thirty years, is never fatal, and in the track of its ulceration there is often new and permanent tissue formed. Whereas, typical tuberculosis may and does attack every tissue of the body, is never local, is short in duration, always fatal, and its ulcers never heal permanently in one of its parts while still progressing in another.

Clinical observations sometimes should have as much weight in classification of diseases, pathologically and etiologically, as the microscope or a remedy. And that when in given diseases, the clinical histories of which are widely diverse, the microscope which only finds one and sometimes two minor and perhaps accidental points of analogy, should make a classification, is not in harmony with the usual law of the greater weight of evidence, establishing a decision. There are tumors which pursue widely different clinical courses, which the microscope cannot foretell positively. The time will doubtless come when it can say that such or such a new formatian or cell is malignant and will recur and produce death, or is harmless in character, but until it does, we let clinical history classify them.

The eye men there are Stellwag and Fuchs, both of whom always make an iridectomy in cataract operations.

Politzer trepans the mastoid cells often for suppurative conditions with the small chisel and mallet, scraping out the diseased cavity with the sharp spoon, as also in Berlin.

There are many Americans in Vienna, and they all, who are studying throat and eye diseases, find in Dr. Frank Sims,

(who once lived in Atlanta, and is a brother-in-law of the late Dr. Bizzell) a most valuable friend. He is an assistant in Prof. Schnitzler's throat, and Prof. Fuchs' eye clinic. The life in Vienna seems to agree with him.

I hope the hasty putting together, and the cursory mention of merely the leading points of some of the notes I have taken during my nearly eight months stay in Europe, as embodied in these notes and the letters I wrote you before, may have been of some interest to your readers.

THOMAS M. MCINTOSH.

